# Nontyphoidal *Salmonella* among Children under 5 Years Old in Sub-Saharan Africa and South Asia in the Global Enteric Multicenter Study

**DOI:** 10.4269/ajtmh.21-0762

**Published:** 2021-11-08

**Authors:** Rina Das, Md. Ahshanul Haque, Mohammod Jobayer Chisti, Tahmeed Ahmed, Abu Syed Golam Faruque

**Affiliations:** Nutrition and Clinical Services Division, International Center for Diarrheal Disease Research, Bangladesh (icddr,b), Dhaka, Bangladesh

## Abstract

Factors associated with nontyphoidal *Salmonella* (NTS) infection have not been well characterized to date. We aimed to compare the associated factors among children under age 5 years with NTS infection in sub-Saharan Africa and South Asia. Data from children having moderate-to-severe diarrhea (MSD) and asymptomatic children with NTS isolated from fecal specimens were extracted from the Global Enteric Multicenter Study (GEMS), conducted from December 2007 to March 2011. Compared with NTS-negative children, NTS-associated MSD cases in South Asia were associated with the presence of goat in the house (adjusted odds ratio [aOR]: 2.15; 95% confidence interval [CI]: 1.25–3.70) and handwashing after handling an animal (aOR: 2.26; 95% CI: 1.36–3.74). In sub-Saharan Africa, children with NTS associated MSD had a greater association with stunting (1.21 95% CI: 1.01–1.45), longer duration of diarrhea (aOR: 1.25 95% CI: 1.19–1.31); presence of cow in house (aOR: 1.54 95% CI: 1.09–2.16), handwashing after handling animal (aOR: 2.41 95% CI: 1.74–3.33). Drinking tube well water (aOR: 0.54 95% CI: 0.32–0.91), availability of toilet facility (aOR: 0.58 95% CI: 0.53–0.65), and handwashing before eating (aOR: 0.76 95% CI: 0.57–1.00) and after defecation (aOR: 0.80 95% CI: 0.69, 0.94) were found to be protective. The differentials between children of both regions having fecal NTS are distinct and underscore the need for policymaking for preventive and control strategies targeting stunted children.

## INTRODUCTION

Nontyphoidal *Salmonella* (NTS) is a Gram-negative bacterium responsible for causing disease in both humans and animals worldwide. On a global scale, approximately 3.4 million cases of NTS infection are detected each year.^1^ The Institute of Health Metrics and Evaluation reported that more than 30,000 child deaths were associated with invasive NTS disease and more than 20,000 of such cases of child mortality in 2017 were from western sub-Saharan Africa.^2^^–^^4^ The majority of illnesses in humans caused by NTS are related to gastrointestinal problems, although uncommonly, it also invades the bloodstream. Infants and young children are more susceptible to NTS infection, making them a high-risk population.^5^ Approximately 5% of the NTS infections occur due to extraintestinal and invasive NTS (iNTS). iNTS found in the bloodstream of children is emerging with new pathogenic features.^6^

There are existing knowledge gaps regarding the epidemiology and prevalence of NTS, including clinical presentation, risk factors, and antimicrobial-resistance trends in sub-Saharan Africa and South Asia.^7^ The only study regarding the estimation of the NTS burden in South Asia indicated an incidence of 470 cases per 100,000 person-years.^8^ NTS infection has been given less attention compared with that of *Salmonella* enterica serovar *Typhi* because of the high incidence of *S*. *Typhi* in this region. A good understanding of the risk factors for NTS may therefore prevent diarrhea leading to serious adverse outcomes in young children.

In this study, we aimed to compare and differentiate between the associated factors of NTS infection among children of two distinctly different geographic regions, sub-Saharan Africa and South Asia. We also intended to describe the associations among their demographic, environmental, and socioeconomic characteristics, and clinical features.

## METHODS

### Study site.

Global Enteric Multicenter Study (GEMS) was a prospective case–control study conducted from December 2007 to March 2011 across four sites in Africa (The Gambia, Kenya, Mali, and Mozambique), and three sites in Asia (Bangladesh, India, and Pakistan).^9^^–^^11^

### Study design and study participants.

GEMS had a well-defined standardized recruitment protocol.^10^ The published,^12^ working hypothesis,^9^ epidemiology,^10^ clinical,^13^ laboratory,^10^ and statistical methods^14^ of GEMS have been described elsewhere.^15^ For our present analysis, we enrolled NTS-positive children from both case and control group of GEMS (*N* = 22,567) because NTS was present in stool among the moderate-to-severe diarrhea (MSD) cases (*N* = 9,439) and healthy controls (*N* = 13,128) of the GEMS. Children having NTS in stool constituted the cases (*N* = 378), and children without NTS in stool were considered as the controls (*N* = 1134). The control children from each study site were randomly selected from the database of enrolled GEMS by computer-based randomization technique using SPSS version 25.0 (SPSS Inc., Chicago, IL) to prevent any selection bias. To substantiate the statistical power of our analyses, we used a site-specific case–control ratio of 1:3. In this data analysis, we include 1,512 (6.7%) children under 5 years of age from GEMS. Of the 378 (1.68%) NTS-positive cases, 190 were MSD cases with 105 (55.26%) in sub-Saharan Africa and 85 (44.74%) in South Asia. The remaining 188 were NTS-positive, asymptomatic healthy children with 92 (48.94%) from sub-Saharan Africa and 96 (51.06%) from South Asia; 1,134 children were NTS-negative controls (Figure 1).

**Figure:**
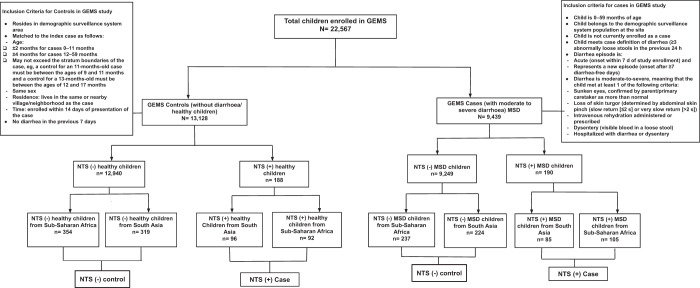
Figure 1. Study profile of enrolled children. GEMS = Global Enteric Multicenter Study; MSD = moderate-to-severe diarrhea; NTS = nontyphoidal *Salmonella.*

### Stool sample collection and fecal microbiology.

Stool specimens were collected from every child at the time of enrollment. Stool samples were processed using the GEMS laboratory procedure protocol.^16^^,^^17^ Bacterial pathogens, viruses, and protozoa were identified using standard laboratory methods.^16^ NTS has been isolated using standard bacteriological methods.^16^ In our analysis, we did not use the TaqMan molecular diagnostic data that included information on isolates of NTS because TaqMan could not differentiate typhoidal and NTS.^18^

### Variable of interest.

Region is categorized as sub-Saharan Africa and South Asia. Clinical manifestations such as vomiting (three or more times/day), fever on admission (temperature measured at least 38°C), and visible blood in stools was assessed based on reports of the primary caretakers.^10^ Diarrhea was defined as the passage of three abnormally loose or watery stools in the previous 24 hours.^10^^,^^19^ Each child was assessed for diarrhea for eligibility of enrollment, and additional criteria for inclusion involved fulfilling at least one of the following conditions for MSD: sunken eyes, loss of skin turgor or very slow, intravenous rehydration administered or prescribed, or admission to hospital with diarrhea or dysentery.^10^ Height, weight, and mid-upper-arm circumference (MUAC) were measured at enrollment and after rehydration for each child.^20^ Details of anthropometry have been described elsewhere.^10^ The height/length-for-age, weight-for-age, and weight-for-height/length z-scores (HAZ, WAZ, and WHZ) have been calculated with a WHO SAS macro using the WHO Child Growth Standards as the reference population.^21^^–^^23^ The interpretation of malnutrition following a WHO guideline was stunted, underweight, and wasted as having HAZ ≤ 2, WAZ ≤ 2, and WHZ ≤ 2. Child breastfeeding status was categorized as breastfed and nonbreastfed.

Sociodemographic information regarding the enrolled children and their household characteristics included mother as a primary caretaker, mother’s education, household size, and building materials of the floor as explanatory variables. Further variables addressed included handwashing practices, use of handwashing material, access to the main source of drinking water, available water treatment method, available toilet facility, and animals on the premises. The households were classified into socioeconomic status quintiles based on the wealth quintile index (poor, lower middle, middle, upper middle, and rich). This was done to assess possible factors associated with disease and as indicators for creating a wealth index for each site.^10^^,^^24^^,^^25^ The stool was examined for consistency and the presence of pus, mucus, red blood cells, and other copathogens.

### Statistical analysis.

The statistical analysis was carried out using version 15.0 of the STATA software (Stata Corp., College Station, TX). Categorical variables were expressed in terms of frequency and percentage, and mean and standard deviation (SD) were used to represent continuous variables. To compare the mean differences, a Student’s *t*-test was conducted for continuous variables, and variations in proportions were compared with the chi-square test. The strength of association was determined by estimating odds ratios (ORs) and 95% confidence intervals (CIs) using simple logistic regression. Initially, we performed bivariate analyses of the relevant characteristics to identify factors that were significantly associated with NTS. Eventually, we performed multiple logistic regression analyses to identify the independently associated factors of NTS infection in children aged < 5 years in both regions (sub-Saharan Africa and South Asia), controlling for the site (country) as a cluster for NTS-positive children having MSD and healthy asymptomatic children. Covariates with a *P* < 0.25 in the bivariate analysis were included in the multiple regression model via a stepwise forward selection technique, as more traditional levels such as *P* < 0.05 can fail in identifying variables known to be of importance,^26^ whereas other relevant variables, such as age and sex, were adjusted for the variable with any *P* value due to their biological and public health importance. All relevant covariates were included in the subsequent model to obtain an adjusted final model. To detect multicollinearity, the variance inflation factor (VIF) was determined, and none of the variables provided a VIF value > 5. Lastly, a *P* value < 0.05 was considered statistically significant in multivariable analysis.

### Ethical consideration.

The study was approved by the ethical committees at the University of Maryland School of Medicine and each of the participating institutes across each of the seven study sites.^10^ Signed informed consent for the enrollment of each child in the study was collected from the parents or legal guardians (for both sick cases and healthy controls).

## RESULTS

### Country-specific baseline characteristics of the NTS-positive under 5 children in South Asia and sub-Saharan Africa.

The baseline characteristics of the study population having NTS-positive stools are presented in the Supplemental Table 1a. We collected data from 1,512 children enrolled in GEMS. Among them, 41.4% were breastfed; 50.3% of children had MSD, and 14.6% reported the presence of blood in the stool. Approximately 97% of the primary caretakers were mothers, and 70% were literate. Only 23% of children belonged to a middle-class family. Sixty-five percent had fowl/rodent and 44% had a cow in the house, and 50% of families indicated the presence of a dog or cat in the house. One-fourth of the households used tube well water as the main source of drinking water. A toilet facility was available in 87% of the houses. Almost 79% used soap and water during handwashing. Handwashing practice was observed among only 13% after handling an animal.

### Characteristic of NTS-positive children with MSD in South Asia and sub-Saharan Africa.

Half (50.6%) of the NTS-positive children suffering from MSD were aged between 0 and 11 months, and overall 42.7% were female in South Asia. Less often, they had sunken eyes and loss of skin turgor as indicators of MSD. More children lived in households with a natural floor made of earth, sand, and dung; had a domestic animal in the household; used tube well water as the main source of drinking water; and practiced handwashing more often before eating and cooking, after defecation, and after handling an animal. However, among caregivers and family members, there was less frequent handwashing practice after cleaning the child and less commonly use water and soap for handwashing. Laboratory examinations reported loose, watery stool with the presence of mucus with *Giardia* as a copathogen (Supplemental Table 1b) less frequently compared with NTS-negative children. Children belonging to both cases and controls were breastfed and used a toilet facility, and the primary caretaker was mostly the mother.

In sub-Saharan Africa, a significantly higher proportion of under 5 children with MSD and NTS-positive stool were stunted and underweight. There were higher durations of diarrhea and more children had the clinical indicators for MSD, but dysentery was less common. The mother was less often the primary caretaker; floors were made of earth, sand, and dung; households had a domestic animal, used non–tube well water as the main source of drinking water, often used treated drinking water. Families less frequently had a toilet facility at the house and were less likely to practice handwashing before eating and after defecation; however, they practiced handwashing more often before nursing the child, after cleaning the child, and after handling animal. On examination stool was loose and watery with fecal mucus compared with stool specimens of the control group. *Campylobacter* and *Giardia* were reported as the more frequent copathogens (Supplemental Table 1b) in the children of the control group compared with children from the NTS-positive group.

### Characteristics of asymptomatic NTS-positive healthy children in South Asia and sub-Saharan Africa.

Compared with asymptomatic NTS-negative children in South Asia, asymptomatic NTS-positive children were more often aged between 0 and 11 months, were more frequently underweight but less often stunted. These aforementioned asymptomatic NTS-positive children had more frequent episodes of fever; were less likely to live in households with floors made of earth, sand, and dung or have a cow at the house; more frequently reported that there was no treatment of drinking water. They also practiced handwashing after defecation more frequently, but were less likely to do so before eating or after handling an animal. *Campylobacter* was detected as a copathogen (Supplemental Table 1b) more often.

In sub-Saharan Africa among NTS-positive cases, more than half of the children were aged 0 to 11 months; they were more likely to be breastfed and malnourished; use tube well water as the main source of drinking water, and have a toilet facility for fecal disposal. Caregivers were less likely to practice handwashing before eating and or nursing a child, but more frequently did so before cooking. Stool samples collected from these children were less commonly mixed with mucus, and *Campylobacter* and *Giardia* were more often isolated among the NTS-negative control group (Supplemental Table 1b).

### Factors associated with MSD among children having NTS present in stool.

In our analysis, it was observed that the NTS-positive children who presented with MSD had greater odds of being from the older age group (adjusted OR [aOR]: 4.36; 95% CI: 2.98–6.38), and the caretakers frequently practiced handwashing after handling domestic animals (aOR: 5.17; 95% CI: 2.41–11.07). These children presented more frequently with fever (aOR: 7.29; 95% CI: 2.89–18.40) and mucus on stool examination (aOR: 5.17; 2.41–11.07); *Giardia* (aOR: 0.27; 95% CI: 0.13–0.56) was observed as a copathogen less often compared with the NTS-positive asymptomatic healthy control children (Table 1).

**Table 1:** Factors associated with MSD among children having NTS present in stool

Characteristics	Unadjusted OR (95% CI)	*P* value*	aOR (95% CI)	*P* value*
Age group (months)				
0–11	Reference			
12–23	1.03 (0.65–1.63)	0.92	1.43 (0.99–2.07)	0.06
24–59	1.77 (1.02–3.06)	0.04	4.36 (2.98–6.38)	< 0.01
Sex				
Boy	Reference			
Girl	1.03 (0.68–1.55)	0.90	1.12 (0.82–1.53)	0.48
Wash hand after handling animal				
No	Reference			
Yes	3.54 (1.78–7.04)	< 0.01	5.17 (2.41–11.07)	< 0.01
*Giardia*				
No	Reference			
Yes	0.45 (0.23–0.82)	< 0.01	0.27 (0.13–0.56)	< 0.01
Fever				
No	Reference			
Yes	7.72 (4.89–12.20)	< 0.01	7.29 (2.89–18.40)	< 0.01
Mucus in stool				
No	Reference			
Yes	14.64 (8.56–25.06)	< 0.01	5.17 (2.41–11.07)	< 0.01

aOR = adjusted odds ratio; CI = confidence interval; MSD = moderate-to-severe diarrhea; NTS = nontyphoidal *Salmonella*; OR = odds ratio.

* *P* values were calculated using multiple logistic regression and all variables are adjusted for age, gender, wealth index, presence of copathogen, and site. Stunting: height/length-for-age z-score ≤ 2, (%; for < 5 years of age).

### Factors associated with NTS among under 5 children in sub-Saharan Africa and South Asia.

In sub-Saharan Africa, NTS-positive MSD children who visited the health facility were more often stunted (aOR: 1.21; 95% CI: 1.01–1.45), had a longer duration of diarrhea (aOR: 1.25; 95% CI: 1.19–0.31), and less often presented with visible blood in stool (aOR: 0.51; 95% CI: 0.44–0.60); loss of skin turgor was more common (aOR: 2.41; 95% CI: 1.74–3.33). They required intravenous rehydration less frequently (0.85; 95% CI: 0.75–0.96) and were less frequently reported to live in houses with a natural floor (aOR: 0.50; 95% CI: 0.28–0.88) or to use tube well water as the main source of drinking water (aOR: 0.54; 95% CI: 0.32–0.91). The presence of a cow at the house was more frequent (aOR: 1.54; 95% CI: 1.09–2.16), and availability of toilet facility was less frequent (aOR: 0.58; 95%: 0.53–0.65). The practice of handwashing was less frequent after defecation (aOR: 0.80; 95% CI: 0.69–0.94) but more frequent after handling an animal (aOR: 2.41; 95% CI: 1.74–3.33). Loose, watery stool (aOR: 1.30; 95% CI: 1.13–1.50) and presence of mucus in stool (aOR: 2.11; 95% CI: 1.39–3.22) was more common, but detection of *Campylobacter* (aOR: 0.54; 95% CI: 0.49–0.61) in stool as a copathogen was less frequently observed (Table 2) compared with NTS-negative MSD children.

**Table 2:** Results of multiple logistic regression in exploring the factors of NTS infection in MSD children aged < 5 years in sub-Saharan Africa and South Asia

Factors	Sub-Saharan Africa				South Asia			
	Unadjusted OR (95% CI)	*P* value*	aOR (95% CI)	*P* value*	Unadjusted OR (95% CI)	*P* value*	aOR (95% CI)	*P* value*
Age group (months)								
0–11	Reference				Reference			
12–23	0.92 (0.54–1.56)	0.75	1.06 (0.78–1.44)	0.70	0.84 (0.46–1.53)	0.57	0.82 (0.31–2.20)	0.69
24–59	1.02 (0.57–1.84)	0.94	1.47 (1.14–1.89)	< 0.01	1.09 (0.58–2.05)	0.79	1.15 (0.18–7.54)	0.89
Gender								
Boy	Reference				Reference			
Girl	0.76 (0.48–1.20)	0.24	0.71 (0.44–1.17)	0.18	0.93 (0.56–1.56)	0.79	0.85 (0.70–1.02)	0.09
Stunting								
No	Reference				Reference			
Yes	1.41 (0.86–2.32)	0.18	1.21 (1.01–1.45)	0.04	1.34 (0.80–2.22)	0.26		–
Clinical features								
Visible blood in the stool								
No	Reference				Reference			
Yes	0.72 (0.35–1.49)	0.37	0.51 (0.44–0.60)	< 0.01	1.23 (0.74–2.03)	0.43		–
Duration of diarrhea (day) on admission	1.32 (0.78–2.21)	0.30	1.25 (1.19–1.31)	< 0.01	0.56 (0.30–1.03)	0.06		–
Indicators of MSD								
Loss of skin turgor								
No	Reference				Reference			
Yes	2.31 (1.41–3.80)	< 0.001	2.41 (1.74–3.33)	< 0.01	0.80 (0.37–1.71)	0.56		–
Required intravenous rehydration								
No	Reference				Reference			
Yes	1.23 (0.71–2.13)	0.46	0.85 (0.75–0.96)	0.01	1.04 (0.49–2.19)	0.92		–
Dysentery								
No	Reference				Reference			
Yes	0.76 (0.34–1.69)	0.50	2.21 (1.39–3.53)	< 0.01	1.11 (0.67–1.83)	0.68		–
Sociodemographic characteristics								
Natural floor								
No	Reference				Reference			
Yes	0.72 (0.45–1.61)	0.18	0.50 (0.28–0.88)	0.02	1.34 (0.85–2.30)	0.19		–
Main source of drinking water								
Non–tube well	Reference				Reference			
Tube well	0.61 (0.22–1.68)	0.34	0.54 (0.32–0.91)	0.02	1.63 (0.98–2.69)	0.06		–
Toilet facility available								
No	Reference				Reference			
Yes	0.66 (0.38–1.18)	0.16	0.58 (0.53–0.65)	< 0.01	1.07 (0.37–3.06)	0.90		–
Animal in house								
Cow								
Absent	Reference				Reference			
Present	1.44 (0.89–2.31)	0.14	1.54 (1.09–2.16)	0.01	2.33 (1.32–4.12)	< 0.01		–
Goat								
Absent	Reference				Reference			
Present	1.22 (0.73–1.95)	0.40		–	2.40 (1.13–5.12)	0.02	2.15 (1.25–3.70)	< 0.01
Handwashing practice								
Wash hands before eating								
No	Reference				Reference			
Yes	0.84 (0.45–1.56)	0.58	0.76 (0.57–1.00)	0.05	2.44 (1.18–5.04)	0.02		–
Wash hands after defecation								
No	Reference				Reference			
Yes	0.77 (0.45–1.32)	0.35	0.80 (0.69–0.94)	< 0.01	1.61 (0.87–2.99)	0.13		–
Wash hands after handling animal								
No	Reference				Reference			
Yes	2.16 (1.03–4.50)	0.04	2.41 (1.74–3.33)	< 0.01	2.44 (1.31–4.57)	< 0.01	2.26 (1.36–3.74)	< 0.01
Laboratory findings								
Stool consistency								
Soft/hard	Reference				Reference			
Loose watery	1.16 (0.73–1.85)	0.53	1.30 (1.13–1.50)	< 0.01	0.77 (0.46–1.31)	0.34		–
Mucus in stool								
No	Reference				Reference			
Yes	1.69 (1.01–2.83)	0.04	2.11 (1.39–3.22)	< 0.01	1.23 (0.55–2.04)	0.41		–
Copathogen isolated in stool								
*Campylobacter *spp.								
Absent	Reference				Reference			
Present	0.54 (0.21–1.56)	0.19	0.54 (0.49–0.61)	< 0.01	0.98 (0.54–1.81)	0.96		–
*Giardia*								
Absent	Reference				Reference			
Present	0.63 (0.31–1.29)	0.21		–	0.49 (0.19–1.22)	0.13	0.55 (0.41–0.72)	< 0.01

aOR = adjusted odds ratio; CI = confidence interval; MSD = moderate-to-severe diarrhea; NTS = nontyphoidal *Salmonella*; OR = odds ratio.

* *P* values were calculated using multiple logistic regression; adjusted for age, gender, and site as a cluster, variables with *P* value < 0.05 were considered for inclusion in the final model.

In Table 3, in sub-Saharan Africa asymptomatic NTS-positive children were more stunted (aOR: 1.10; 95%CI: 0.66–1.83) and more likely to use a toilet facility for fecal disposal (1.29; 95% CI: 1.12–1.48); however, handwashing before eating (0.78; 95% CI: 0.61–0.99) and before nursing a child (0.66; 95% CI: 0.49–0.88) was less frequent. Presence of mucus in stool was less common (0.51; 95% CI: 0.46–0.56) and *Campylobacter* (0.74; 95% CI: 0.70–0.78) and *Giardia* (0.54; 95% CI: 0.35–0.83) were less commonly isolated as copathogens. In South Asia, asymptomatic NTS-positive healthy children lived in households with fewer under 5 children (aOR: 0.88; 95% CI: 0.75–0.87); reported the presence of cow as a domestic animal less often (aOR: 0.81: 95% CI: 0.75–0.87); less frequently practiced handwashing after handling an animal (aOR: 0.63; 95% CI: 0.49–0.81), and *Campylobacter* was isolated more often (aOR: 2.92; 95% CI: 2.19–2.89) as a co-pathogen compared with asymptomatic NTS-negative children.

**Table 3:** Results of multiple logistic regression in exploring the factors of asymptomatic NTS-positive children aged < 5 years in sub-Saharan Africa and South Asia

Factors	Sub-Saharan Africa				South Asia			
	Unadjusted OR (95% CI)	*P* value*	aOR (95% CI)	*P* value*	Unadjusted OR (95% CI)	*P* value*	aOR (95% CI)	*P* value*
Age group (months)								
0–11	Reference				Reference			
12–23	0.59 (0.36–0.98)	0.05	0.56 (0.33–0.95)	0.03	0.66 (0.39–1.11)	0.12	0.53 (0.47–0.60)	< 0.01
24–59	0.28 (0.14–0.57)	< 0.01	0.27 (0.17–0.43)	< 0.01	0.47 (0.26–0.86)	0.01	0.44 (0.36–0.55)	< 0.01
Sex								
Boy	Reference				Reference			
Girl	1.09 (0.69–1.75)	0.69	1.06 (0.82–1.37)	0.66	0.68 (0.43–1.09)	0.11	0.64 (0.50–0.82)	< 0.01
Stunting								
No	Reference				Reference			
Yes	1.10 (0.66–1.83)	0.71	1.42 (1.01–1.81)	< 0.01	0.78 (0.47–1.27)	0.31	–	
Sociodemographic characteristics								
Children < 5 years in house	1.37 (0.75–2.48)	0.30		–	0.98 (0.59–1.63)	0.94	0.88 (0.75–0.87)	< 0.01
Cow in house								
Absent	Reference				Reference			
Present	1.07 (0.67–1.70)	0.78		–	0.78 (0.43–1.43)	0.42	0.81 (0.75–0.87)	< 0.01
Main source of drinking water								
Non–tube well	Reference				Reference			
Tube well	0.87 (0.39–1.94)	0.73		–	0.57 (0.34–0.94)	0.03		–
Toilet facility available								
No	Reference				Reference			
Yes	1.33 (0.72–2.45)	0.36	1.29 (1.12–1.48)	< 0.01	0.89 (0.28–2.85)	0.86		–
Handwashing practice									
Wash hands before eating								
No	Reference				Reference			
Yes	0.77 (0.39–1.52)	0.46	0.78 (0.61–0.99)	0.04	0.62 (0.38–0.99)	0.05		–
Wash hands before nursing child								
No	Reference				Reference			
Yes	0.81 (0.49–1.37)	0.44	0.66 (0.49–0.88)	< 0.01	1.21 (0.74–1.99)	0.44		–
Wash hands after handling animal								
No	Reference				Reference			
Yes	0.43 (0.15–1.24)	0.12		–	0.62 (0.28–1.36)	0.23	0.63 (0.49–0.81)	< 0.01
Laboratory findings									
Mucus in stool								
No	Reference				Reference			
Yes	0.62 (0.25–1.53)	0.30	0.51 (0.46–0.56)	< 0.01	1.05 (0.57–1.94)		0.87		–
Copathogen isolated in stool									
*Campylobacter *spp.								
Absent	Reference				Reference			
Present	0.72 (0.35–1.49)	0.38	0.74 (0.70–0.78)	< 0.01	2.45 (1.45–4.12)	< 0.01	2.92 (2.19–2.89)	< 0.01
*Giardia*								
Absent	Reference				Reference			
Present	0.43 (0.23–0.81)	0.01	0.54 (0.35–0.83)	< 0.01	1.07 (0.62–1.87)	0.80		–

aOR = adjusted odds ratio; CI = confidence interval; MSD = moderate-to-severe diarrhea; NTS = nontyphoidal *Salmonella*; OR = odds ratio.

* *P* values were calculated using multiple logistic regression; adjusted for age, gender, wealth index, presence of copathogen and site as cluster; variables with *P* value < 0.05 were considered for inclusion in the final model. Stunting: height/length-for-age z-score ≤ 2 (%; for < 5 years of age).

Irrespective of MSD, when we considered both the regions combined, we found that all NTS-positive children who either presented with MSD or as asymptomatic had greater odds of being stunted (aOR: 1.27; 95% CI: 1.05–1.53), and the mother was less often the primary caretaker of the child (aOR: 0.62; 95% CI: 0.51–0.75). Among all these NTS-positive children in both region irrespective of MSD, a goat present at the household as a domestic animal was more common (aOR: 1.23; 95% CI: 1.03–1.48), clinically presented with fever (aOR: 1.40; 95% CI: 1.03–1.89), mucus present in stool (aOR: 1.22; 95% CI: 1.07–1.40] and *Giardia* (aOR: 0.67; 95% CI: 0.49–0.91) was less often observed as a copathogen (Table 4).

**Table 4:** Results of multiple logistic regression in exploring the factors of NTS infection in children aged < 5 years in both sub-Saharan Africa and South Asia (both MSD and asymptomatic NTS)

Characteristics	Unadjusted OR (95% CI)	*P* value*	aOR (95% CI)	*P* value*
Age group (months)				
0–11	Reference			
12–23	0.73 (0.56–0.95)	0.02	0.75 (0.57–0.99)	0.04
24–59	0.62 (0.45–0.84)	< 0.01	0.64 (0.44–0.92)	0.02
Gender				
Boy	Reference			
Girl	0.86 (0.68–1.09)	0.22	0.85 (0.74–0.98)	0.03
Stunting				
No	Reference			
Yes	1.12 (0.88–1.44)	0.36	1.27 (1.05–1.53)	0.02
Sociodemographic characteristics				
Primary care taker				
Other family member	Reference			
Mother	0.81 (0.40–1.66)	0.57	0.62 (0.51–0.75)	< 0.01
Animal present at house				
Goat				
No	Reference			
Yes	1.19 (0.94–1.52)	0.15	1.23 (1.03–1.48)	0.03
Clinical feature				
Fever				
No	Reference			
Yes	1.54 (1.22–1.95)	< 0.01	1.40 (1.03–1.89)	0.03
Stool examination				
Mucus				
No	Reference			
Yes	1.44 (1.13–1.83)	< 0.01	1.22 (1.07–1.40)	< 0.01
Copathogen				
*Giardia*				
Absent	Reference			
Present	0.61 (0.44–0.85)	< 0.01	0.67 (0.49–0.91)	0.01

aOR = adjusted odds ratio; CI = confidence interval; MSD = moderate-to-severe diarrhea; NTS = nontyphoidal *Salmonella*; OR = odds ratio.

* *P* values were calculated using multiple logistic regression and all variables are adjusted for age, gender, wealth index, presence of co pathogen and site. Stunting: height/length-for-age z-score ≤ 2 (%; for < 5 years of age).

## DISCUSSION

In our study, approximately half of the NTS cases were children aged between aged 0 and 11 months in both distinct geographic regions. Our findings support previous studies indicating that young children, particularly infants, are more susceptible to NTS infection.^27^^,^^28^

Compared with South Asia, stunting was significantly associated with both symptomatic and asymptomatic NTS infections among children from sub-Saharan Africa. The previous study from Africa reported malnutrition to be a risk factor for NTS infection.^29^ We assume that many factors may be attributable to the discrepancy in findings between sub-Saharan Africa and South Asia, including lower levels of the immunocompromised children in South Asia, owing to fewer cases with advanced HIV infection and a lower burden of various comorbidities, including malaria. Studies have indicated that NTS infection is more often invasive in immunocompromised patients compared with healthy patients. The role of the interleukin-17 (IL-17) in NTS infection has been suggested to increase vulnerability to invasive NTS infection; this is being documented by human studies with IL-12p40 (inherently agnostic cytokine) deficiency,^30^ suggesting that IL-12 can play a critical role in protecting against NTS. There is reduced IL-12 expression in malnourished children compared with well-nourished controls,^31^ which may make them more vulnerable to NTS infection.

In sub-Saharan Africa, the increased duration of diarrhea is a commonly accompanying clinical feature among NTS-positive children. In South Asia, we did not find any association between NTS-positive MSD and the duration of diarrhea. Case–control studies from the United States,^32^ Palestine,^33^ Denmark,^34^ and Brazil^35^ have reported an association with acute diarrhea and NTS, whereas no such association was found in Thailand^36^ or Bangladesh.^37^ The duration of diarrhea was not associated with NTS in South Asia, probably due to the low prevalence of HIV.^38^

In sub-Saharan Africa, caregivers had fewer complaints about visible blood in the stool. No association was found with NTS-positive MSD and visible blood in stool in South Asia. Such observations are concomitant with the findings of other studies.^39^^,^^40^ Similarly, dysentery as a criterion suggesting MSD was significantly related to NTS infection in sub-Saharan Africa but not in South Asia. Immunocompromised persons, including those infected with HIV and malaria, and infants and young adults living in areas where malnutrition is widespread are particularly at risk of acquiring iNTS disease.^41^^–^^43^ The low prevalence of HIV and malaria in South Asia is a likely explanation for the absence of any significant association between dysentery and NTS infection. Since 1966, NTS has been reported as a cause of bacteremia in 33 of 54 African countries, spanning the five geographic areas of Africa, but mainly in sub-Saharan Africa, according to a systematic review.^42^ However, data suggest that iNTS is a significantly less serious concern in Asia than typhoid fever. The high frequency of falciparum malaria in Africa could be one explanation for this discrepancy between continents.^44^

In our research, we observed that NTS isolation is associated with an increased need for IV rehydration, and there is evidence of increased inflammation of the gut, including higher levels of stool mucus in sub-Saharan Africa. These observations are suggestive of the pathogenicity of NTS causing secretory diarrhea, resulting in a fluid-electrolyte deficit in the study children; these findings are consistent with a study conducted in Bangladesh.^45^ In sub-Saharan Africa, NTS-positive children had a frequent loss of skin turgor, which was indicative of dehydration and the need for oral rehydration therapy instead of intravenous saline infusions.

We also report that children with MSD and associated NTS infection were more likely to drink tube well water in sub-Saharan Africa. In South Asia, NTS was not linked with treatment of drinking water at the home. Previous findings from another study in Asia indicated that the type of water was not a risk factor for NTS infection.^45^ A research in Bangladesh found that patients with *S. typhi* bacteremia have a greater likelihood of unboiled water intake than those with NTS bacteremia.^46^ This does not rule out the remote possibility of using untreated water by NTS-positive children.

In Africa, mothers’ handwashing before food preparation, after defecation, and before eating were correlated with less diarrhea.^47^ Different studies have shown that handwashing before eating has a positive impact on preventing shigellosis^48^ and improved water, sanitation, and hygiene reduced childhood diarrhea.^49^ In our study, we also found primary caretakers’ handwashing before eating and after defecation had a protective effect on MSD children with NTS infection in sub-Saharan Africa. There was no association between handwashing practices before eating and NTS in South Asia, perhaps because handwashing practice before eating was much lower than after defecation.^50^ In this region, handwashing practices with soap and water are poor, but most women from slums and rural areas rub their hands on the ground or use soil and rinse them with water after defecation during handwashing.^51^ Practicing handwashing after handling animals was significantly associated with NTS-associated MSD in children in both regions, probably for the same reason as the mothers washing hands only with water but not soap after handling animals. In our study, we found no association between improved sanitation and NTS in South Asia but had a protective effect in sub-Saharan Africa. In South Asia, gross diversity in access to improved sanitation progress is prevalent.^52^

In sub-Saharan Africa, NTS-positive cases were significantly associated with the presence of a cow or goat in South Asia, but the nature of the role of animals in the transmission of NTS is not clear. However, research at the GEMS site in Kenya found that a moderately high proportion of fecal chicken pools were positive for NTS.^53^ Among the asymptomatic NTS-positive children, presence of a cow was found to be protective in South Asia. Such evidence make the interaction between NTS and domestic animals at home an interesting topic for future studies.

NTS cases often had stools with mucus in sub-Saharan Africa, but among asymptomatic children, mucus was found less often in the stool. There have been reports of elevated intestinal inflammation, including higher levels of mucus and red blood cells. These data may suggest invasive pathogenicity of NTS in children under 5 years of age, which is consistent with other studies.^45^

We found that *Giardia* was less likely to be observed as a copathogen among NTS-positive children in sub-Saharan Africa and South Asia. Conversely, in South Asia *Campylobacter* was significantly associated with NTS-positive cases. In other studies, the prevalence and concentration of *Campylobacter* in household pets was lower than that of farm animals that did not come in contact with the public.^54^ *Campylobacter* was highly associated with pets.^55^ A hospital-based surveillance study reported a lack of association of *Campylobacter* and *Giardia* as copathogens with NTS cases in Bangladesh.^45^ Additionally, we did not find any association of NTS infection with the primary caretaker’s education, socioeconomic context, handwashing with soap, and overcrowding at the household level.

The strengths of our study involved unbiased sampling, a large sample size, and high-quality standard laboratory performance. The study looked for the factors associated with both symptomatic and asymptomatic NTS infection among children under 5 years from both regions, which further enriches the findings of the study.

Our study also has some limitations. We could not determine the effect of HIV and factors related to HIV infection, and other associated factors such as HIV data in children were absent for South Asia. We assume that a stronger predisposing factor for NTS could be immunocompromised status (e.g., HIV infection) of children with severe malnutrition, which we could not explore in our analysis.

## CONCLUSION

Distinct differences in sociodemographic and clinical characteristics among children with NTS between South Asia and sub-Saharan Africa were observed. Further studies are required to explore diversity within and among regions and emphasize the importance of policy-making for the prevention of nutritional disorders including stunting among children under 5 years in sub-Saharan Africa and South Asia.

## Supplemental Material


Supplemental materials
