# Supplementary material for: Nontyphoidal Salmonella among Children under 5 Years Old in Sub-Saharan Africa and South Asia in the Global Enteric Multicenter Study
Source: Am J Trop Med Hyg. 2021 Nov 8;106(2):504–12. doi: 10.4269/ajtmh.21-0762 (PMC8832896; doi:10.4269/ajtmh.21-0762)
Supplement: Supplementary file 1 [file tpmd210762.SD1.pdf]

Supplementary Tables

Table S1 (a). Country specific baseline characteristics of the NTS-positive children (n=1512)

	South Asia				sub-Saharan Africa			
**Characteristics**	**Bangladesh**	**India**	**Pakistan**	**The Gambia**	**Mali**	**Mozambique**	**Kenya**	**Overall**
**n (%)**	**n = 284**	**n = 72**	**n = 365**	**n = 168**	**n = 8**	**n = 28**	**n = 584**	**n = 1512**
**Non-typhoidal *Salmonella* (+)**	71 (18.8)	18 (4.8)	92 (24.3)	42 (11.1)	2 (0.5)	7 (1.9)	146 (38.6)	378 (25)
**Age group**								
0–11 months	41 (57.8)	11(61.1)	39 (42.4)	19 (45.2)	-	6 (85.7)	68 (46.6)	184 (48.7)
12–23 months	16 (22.5)	3 (16.7)	33 (35.9)	19 (45.2)	1 (50)	1 (14.3)	46 (31.5)	119 (38.5)
24–59 months	14 (19.7)	4 (22.2)	20 (21.7)	4 (9.5)	1 (50)	-	32 (21.9)	75 (19.8)
**Gender (Female)**	29 (40.9)	3 (16.7)	36 (39.1)	24 (57.1)	1 (50)	2 (28.6)	57 (3.0)	152 (40.2)
Breastfed	60 (84.5)	17(94.4)	49 (53.3)	33 (78.6)	-	6 (85.7)	105 (71.9)	270 (71.4)
**Baseline Anthropometry**								
MUAC, median ¶	13.8 ± 1.3	14.2 ± 1.3	13.1 ± 1.4	13.8 ± 1.4	14.6 ± 2.4	13.2 ± 1.5	14.0 ± 1.6	13.7 ± 1.5
HAZ ¶	−1.09 ± 1.1	−1.09 ± 1.2	−2.18 ± 1.4	−1.41 ± 1.8	−1.47 ± 2.4	−1.95 ± 1.6	−1.39 ± 1.4	−1.52 ± 1.4
WAZ ¶	−1.32 ± 1.3	−1.05 ± 1.4	−2.15 ± 1.3	−1.36 ± 1.5	−1.87 ± 2.0	−1.87 ± 1.5	−1.18 ± 1.4	−1.47 ± 1.4
WHZ ¶	−0.95 ± 1.3	−0.63 ± 1.2	−1.33 ± 1.3	−0.77 ± 1.8	−1.69 ± 1.3	−0.93 ± 1.0	−0.58 ± 1.4	−0.87 ± 1.4
**Clinical features**								
Diarrhea	42 (59.2)	4 (22.2)	39 (42.4)	17 (40.5)	2 (100)	5 (71.4)	81(55.5)	190 (50.3)
Visible blood in stool	34 (47.9)	1 (5.6)	9 (9.9)	2 (4.8)	-	1 (14.3)	8 (5.5)	55 (14.6)
Fever	33 (46.5)	7 (38.9)	34 (36.9)	9 (21.4)	1 (50)	3 (42.7)	103(70.6)	190 (50.3)
Vomiting ≥3 times/day	6 (8.5)	3 (16.7)	17 (18.5)	10 (23.8)	1 (50)	-	47 (32.2)	84 (22.2)
**Socio-demographic characteristics**								
Primary caretaker (mother)	71 (100)	18 (100)	88 (95.7)	40 (95.2)	2 (100)	7 (100)	141 (96.6)	367 (97.1)
Mother's education (literate)	66 (92.9)	14 (77.8)	30 (32.6)	38 (90.5)	1 (50)	7 (100)	144 (98.6)	266 (70.4)
People regularly sleep in the house ¶	5.3 ± 2.1	4.9 ± 2.1	8.7 ± 4.8	24.7 ± 18.3	16.5 ± 0.7	5.8 ± 2.9	4.7 ± 1.7	8.1 ±9.1
Presence of under 5 children in the house ¶	0.7 ± 0.6	0.3 ± 0.6	0.9 ± 1.3	1.9 ± 2.9	1.5 ± 0.7	0.9 ± 0.7	0.9 ± 0.9	0.9 ±1.4
**Wealth index**								
poorest	14 (19.7)	5 (27.8)	10 (10.9)	12 (28.6)	-	1 (14.3)	20 (13.7)	62 (16.4)
Lower middle	13 (18.3)	3 (16.7)	25 (27.2)	8 (19.1)	-	2 (28.6)	33 (22.6)	84 (22.2)
Middle	16 (22.5)	3 (16.7)	24 (26.1)	7 (16.7)	-	2 (28.6)	37 (25.3)	89 (23.5)
Upper middle	14 (19.7)	5 (27.8)	15 (16.3)	9 (21.4)	1 (50)	1 (14.3)	18 (12.3)	63 (16.7)
Richest	14 (19.7)	2 (11.1)	18 (19.6)	6 (14.3)	1 (50)	1 (14.3)	38 (26.0)	80 (21.2)
**Animal present at the house**								
Sheep	2 (2.8)	-	-	31 (73.8)	1 (50)	-	46 (31.5)	80 (21.2)
Goat	11 (15.5)	7 (38.9)	8 (8.7)	30 (71.4)	-	1 (14.3)	88 (60.3)	145 (38.4)
Cow	39 (54.9)	1 (5.6)	4 (4.4)	16 (38.1)	-	-	107 (23.3)	167 (44.2)
Fowl/ Rodent	41 (57.8)	9 (50)	13 (14.1)	38 (90.5)	-	4 (57.1)	141 (96.6)	246 (65.1)
Cat	63 (88.7)	17 (94.4)	8 (8.7)	7 (16.7)	-	-	101 (69.2)	196 (51.9)
Dog	60 (84.5)	18 (100)	3 (3.3)	13(30.9)	-	-	103 (70.6)	197 (52.1)

**Main source of drinking water**								
Use tube well water	68 (95.8)	-	-	8 (19.0)	-	-	5 (3.4)	81(21.4)
Use a water treatment method	1 (1.5)	11 (61.1)	31 (36.1)	13 (36.1)	-	-	88 (61.9)	144 (40.1)
**Fecal disposal**								
Toilet facility available in the house	65 (91.6)	18 (100)	89 (96.7)	42 (100)	2 (100)	7 (100)	107 (73.3)	330 (87.3)
**Hand wash material**								
**Routinely hand wash with water and soap**	52 (73.2)	7 (38.9)	60 (65.2)	32 (76.2)	2 (100)	6 (85.7)	139 (95.2)	298 (78.8)
**Practice handwashing**								
Before nursing a child	22 (30.9)	14 (77.8)	32 (34.8)	14 (33.3)	1 (50)	5 (71.4)	40 (27.4)	128 (33.9)
After handling animal	22 (30.9)	-	8 (8.7)	8 (19.1)	-	-	11 (7.5)	49 (12.9)
After cleaning the child	22 (30.9)	15 (83.3)	54 (58.7)	36 (85.7)	-	1 (14.3)	38 (26.0)	166 (43.9)
**Co-pathogens isolated in stool**								
Campylobacter	16 (22.5)	1 (5.6)	32 (34.8)	1 (2.4)	-	-	15 (10.3)	65 (17.2)
Giardia	5 (7.0)	4 (22.2)	18 (19.6)	3 (7.1)	1 (50)	1 (14.3)	19 (13.0)	51 (13.6)
Cryptosporidium	5 (7.0)	0	8 (8.7)	3 (7.1)	0	1 (14.3)	9 (6.2)	26 (6.8)
EAEC	17 (23.9)	5 (27.8)	16 (17.4)	13 (30.9)	0	1 (14.3)	26 (17.8)	78 (20.6)
ETEC	2 (2.8)1	1 (5.6)	9 (9.8)	7 (16.7)	0	1 (14.3)	10 (6.9)	30 (7.9)

¶ Mean ± standard deviation; height-for-age z-score: HAZ, weight-for-age z-score: WAZ, weight-for-height z-score: WHZ, MUAC (mid upper arm circumference): median (mean; for children <5 years of age) mid-upper arm circumference, diarrhea: 3 or more stool/day, fever: measured at least 38-degree Celsius. NTS, non-typhoidal Salmonella, WASH: water, sanitation, and hygiene, EAEC: Enteroaggregative E. coli, ETEC: Enterotoxigenic E. coli

Table S1 (b). Characteristics of the NTS-positive and NTS-negative under 5 sub-Sahara African and South Asian children

		NTS-positive MSD							Asymptomatic NTS-Positive					
		sub-Saharan Africa			South Asia				sub-Saharan Africa			South Asia		
**Characteristics**	**Total NTS (+) MSD n= 190 (%)**	**NTS (+) n= 105 (%)**	**NTS (-) n= 237 (%)**	**p-value***	**NTS (+) n= 85 (%)**	**NTS (-) n= 224 (%)**	**p-value***	**Total Asymptomatic NTS (+) n=188 (%)**	**NTS (+) n=92 (%)**	**NTS (-) n=354 (%)**	**p-value***	**NTS (+) n=96 (%)**	**NTS (-) n=319 (%)**	**p-value***
**Age group**														
0-11m	87 (45.8)	44 (41.9)	7 (40.9)	-	43 (50.6)	110 (49.1)	-	97 (51.60)	49 (53.26)	122 (34.46)	-	48 (50.00)	116 (36.36)	-
12-23m	57 (30.0)	35 (33.3)	4 (35.4)	0.39	22 (25.9)	67 (29.9)	0.75	62 (32.98)	32 (34.78)	134 (37.85)	<0.01	30 (31.25)	110 (34.48)	<0.01
24-59m	46 (24.2)	26 (24.8)	6 (23.6)	0.80	20 (23.5)	47 (20.9)	0.92	29 (15.43)	11 (11.96)	98 (27.68)	<0.01	18 (18.75)	93 (29.15)	<0.01
**Gender (female)**	77 (40.5)	45 (42.7)	118 (49.8)	0.20	88 (39.3)	32 (37.7)	0.46	75 (39.89)	39 (40.39)	142 (40.11)	0.35	36 (37.50)	149 (46.71)	0.01
**Breastfeed**	130 (68.4)	69 (65.7)	163 (68.8)	0.52	61 (71.8)	172 (76.8)	0.37	40 (74.47)	75 (81.52)	227 (64.12)	<0.01	65 (67.71)	223 (69.91)	0.68
**Anthropometry**														
Wasted	41 (21.7)	18 (17.1)	34 (14.4)	0.54	23 (27.4)	62 (27.8)	0.67	24 (12.83)	11 (11.96)	22 (6.21)	<0.01	13 (13.68)	32 (10.06)	0.36
Stunted	72 (38.1)	35 (33.3)	62 (26.2)	<0.01	37 (44.1)	83 (37.1)	0.61	56 (29.95)	27 (29.35)	97 (27.40)	0.19	29 (30.53)	115 (36.16)	<0.01
Underweight	76 (40.0)	39 (37.1)	60 (25.3)	0.01	37 (43.5)	96 (42.9)	0.94	50 (26.60)	18 (19.57)	49 (13.84)	0.22	32 (33.33)	98 (30.72)	0.17
MUAC[¶]	13.6±1.6	13.9±1.5	14.0±1.6	0.51	13.2±1.5	13.8±1.6	0.39	13.89±1.39	14.05±1.54	14.48±1.31	0.13	13.75±1.22	13.87±1.39	0.48
**Clinical features**														
H/o duration of diarrhea[¶]	2.9±1.29	3.1 ±1.38	2.7±1.21	<0.01	2.7 ±1.14	2.9±1.38	0.09	-	-	-		-	-	
Visible blood in stool	52 (27.4)	11 (10.5)	33 (13.9)	0.07	41 (48.2)	98 (43.8)	0.36	3 (1.60)	0 (0.0)	1 (0.28)	ND	3 (3.13)	2 (0.63)	ND
Vomiting ≥3 times/ day	81(42.6)	57 (54.3)	116 (48.9)	0.30	24 (28.2)	64 (28.6)	0.94	3 (1.60)	6 (1.69)	1 (1.09)	ND	2 (2.08)	8 (2.51)	ND
Fever on admission	140 (73.7)	82 (78.1)	178 (75.1)	0.35	58 (68.2)	132 (58.9)	0.20	50 (26.60)	34 (36.96)	102 (28.81)	0.31	16 (16.67)	37 (11.60)	0.07
**Indicators of MSD**														
Sunken eyes	146 (76.8)	102(97.1)	226 (95.4)	0.08	44 (51.8)	135 (60.3)	0.04	0	-	-		-	-	
Loss of skin turgor	52 (27.4)	42 (40.0)	53 (22.4)	<0.01	10 (11.8)	32 (14.3)	0.21	0	-	-		-	-	
IV rehydration needed	36 (18.9)	25 (23.8)	48 (20.3)	<0.01	11 (12.9)	28 (12.5)	0.79	0	-	-		-	-	
Dysentery	48 (25.3)	9 (8.6)	26 (10.9)	0.14	39 (45.9)	97 (43.3)	0.72	0	-	-		-	-	
Required hospital admission	32 (16.8)	23 (21.9)	47 (19.8)	0.19	9 (10.6)	25 (11.2)	0.90	0	-	-		-	-	
**Socio-demographic features**														
Primary caretaker (mother)	183 (96.3)	98 (93.3)	231 (97.5)	0.15	85 (100)	220 (98.2)	ND	184 (97.87)	92 (100)	340 (96.05)	ND	92 (95.83)	316 (99.06)	ND
Mothers' education (Illiterate)	51 (26.84)	20 (19.1)	45 (19.2)	0.97	31 (36.5)	94 (41.9)	0.44	61 (32.45)	21 (22.83)	82 (23.23)	0.75	40 (41.67)	138 (43.26)	0.68

People regularly live in house¶	8.01±7.59	8.85±9.50	9.24±10.53	0.45	6.98±3.97	7.83±4.42	0.02	8.79±10.19	10.61±13.69	12.29±16.18	0.52	7.05±4.30	7.24±4.18	0.63
People regularly sleep in house¶	7.68±7.67	8.30±9.68	9.14±12.40	0.42	6.91±3.93	8.01±5.78	<0.01	8.49±10.27	9.99±13.89	11.68±16.2	0.51	7.05±4.30	7.23±4.19	0.63
Presence of under 5 children at house¶	1.94±1.44	2.15±1.73	2.42±2.03	0.43	1.68±0.92	1.92±1.26	0.07	2.24±1.67	2.76±2.10	2.79±2.59	0.93	1.74±0.87	1.82±1.05	0.16
Natural floor (made of earth, sand, dung)	108 (56.8)	63 (60.0)	160 (67.5)	0.08	45 (52.9)	100 (44.6)	0.19	98 (52.13)	60 (65.22)	226 (63.84)	0.30	38 (39.58)	158 (49.53)	<0.01
Uses tube well water	47 (24.7)	5 (4.8)	18 (7.6)	<0.01	42 (49.4)	84 (37.5)	<0.01	67 (35.64)	43 (46.74)	147 (41.53)	<0.01	24 (25.00)	73 (22.88)	0.58
Treat drinking water	84 (44.2)	64 (60.9)	137 (57.8)	0.25	20 (23.5)	69 (30.8)	0.30	34 (18.09)	8 (8.70)	35 (8.89)	00.54	26 (27.08)	126 (39.50)	<0.01
Toilet facility available	161 (84.7)	81 (77.1)	198 (83.5)	<0.01	80 (94.1)	210 (93.8)	0.92	169 (89.89)	77 (83.70)	281 (79.38)	<0.01	92 (95.83)	307 (96.24)	0.71
**Wealth index**														
Poorest	26 (13.7)	13 (12.4)	37 (15.6)		13 (15.3)	43 (19.2)	-	36 (19.15)	20 (21.74)	77 (21.75)	-	16 (16.67)	54 (16.93)	-
Lower middle	44 (23.2)	23 (21.9)	59 (24.9)	0.05	21 (24.7)	46 (20.5)	<0.01	40 (21.28)	20 (21.74)	72 (20.34)	0.87	20 (20.83)	65 (20.38)	0.95
Middle	45 (23.7)	23 (21.9)	62 (26.2)	0.63	22 (25.9)	56 (25.0)	0.23	44 (23.40)	23 (25.00)	82 (23.16)	0.82	21 (21.88)	78 (24.45)	0.82
Upper middle	34 (17.9)	20 (19.1)	36 (15.2)	0.27	14 (16.5)	40 (17.9)	0.24	109 (15.43)	9 (9.78)	50 (14.12)	0.32	20 (20.83)	59 (18.50)	0.73
Richest	41 (21.6)	26 (24.8)	43 (18.1)	<0.01	15 (17.7)	39 (17.4)	0.65	136 (20.21)	20 (21.74)	73 (20.62)	0.91	19 (19.79)	63 (19.75)	0.98
**An animal in the house**														
Cow	96 (50.5)	68 (64.8)	133 (56.1)	<0.01	28 (32.9)	39 (17.4)	<0.01	71 (37.77)	55 (59.78)	206 (58.19)	0.31	16 (16.67)	65 (20.38)	<0.01
Dog	105 (55.3)	65 (61.9)	137 (57.8)	0.18	40 (47.1)	96 (42.9)	0.09	92 (48.94)	51 (55.43)	192 (54.24)	0.71	41 (42.71)	132 (41.38)	0.79
Cat	100 (52.6)	57 (54.3)	132 (55.7)	0.74	43 (50.6)	96 (42.9)	0.02	96 (51.06)	51 (55.43)	190 (53.67)	0.33	45 (46.88)	136 (42.63)	0.24
Rodent/Fowl	96 (50.5)	54 (51.4)	125 (52.7)	0.49	42 (49.4)	97 (43.3)	0.30	90 (47.87)	51 (55.43)	189 (53.39)	0.78	39 (40.63)	133 (41.69)	0.79
Goat	78 (41.1)	64 (60.9)	133 (56.1)	<0.01	14 (16.5)	17 (7.6)	<0.01	67 (35.64)	55 (59.78)	203 (57.34)	0.43	12 (12.50)	36 (11.29)	0.84
**Hand washing practice**														
Before eating	162 (85.3)	87 (82.9)	202 (85.2)	<0.01	75 (88.2)	169 (75.5)	<0.01	139 (73.94)	79 (85.87)	314 (88.70)	0.07	60 (62.50)	233 (73.04)	0.02
Before cooking	106 (55.8)	104 (43.9)	46 (43.8)	0.96	60 (70.6)	146 (65.2)	0.07	114 (60.64)	47 (51.09)	165 (46.61)	0.25	67 (69.79)	240 (75.24)	0.62
Before nurse/prepare baby food	73 (38.4)	36 (34.3)	70 (29.5)	0.11	37 (43.5)	111 (49.6)	0.37	55 (29.26)	24 (26.09)	107 (30.23)	0.24	31 (32.29)	90 (28.21)	0.46
After defecation	147 (77.4)	78 (74.3)	187 (78.9)	0.01	69 (81.2)	163 (72.8)	0.07	120 (63.83)	64 (69.57)	249 (70.34)	0.81	56 (58.33)	179 (56.11)	0.07
After handling animals	37 (19.5)	15 (14.3)	17 (7.2)	<0.01	22 (25.9)	28 (12.5)	<0.01	12 (6.38)	4 (4.35)	34 (9.60)	ND	8 (8.33)	41 (12.85)	<0.01
After cleaning a child	86 (45.3)	41 (39.1)	77 (32.5)	0.03	45 (52.9)	132 (58.9)	0.22	80 (42.55)	34 (36.96)	131 (37.01)	0.94	46 (47.92)	125 (39.18)	0.28
**Hand wash material**														
Routinely uses water and soap	150 (78.9)	93 (88.6)	212 (89.5)	0.81	57 (67.1)	171 (76.3)	<0.01	148 (78.72)	86 (93.48)	320 (90.40)	0.47	62 (64.58)	230 (72.10)	0.27
**Stool examination**														

Loose watery stool	89 (46.8)	61 (58.1)	129 (54.4)	<0.01	28 (32.9)	87 (38.8)	0.21	0	0	12 (3.39)	ND	0	0	ND
RBC present in stool	28 (14.7)	5 (4.8)	8 (3.4)	ND	23 (27.1)	58 (25.9)	0.75	1	1	0	ND	0	1	ND
Pus present in stool	13 (6.8)	6 (5.7)	15 (6.4)	0.81	7 (8.2)	22 (9.8)	0.61	3 (1.60)	1 (1.09)	3 (0.85)	ND	2 (2.08)	7 (2.19)	ND
Mucus present in stool	125 (66.1)	78 (75.0)	151 (63.9)	0.01	47 (55.3)	112 (50.0)	0.10	22 (11.76)	6 (6.59)	36 (10.17)	<0.01	16 (16.67)	51 (15.99)	0.83
**Co pathogen isolated in stool**														
Campylobacter spp.	24 (12.6)	6 (5.7)	24 (10.1)	<0.01	18 (21.2)	48 (21.4)	0.97	41 (21.81)	10 (10.87)	51 (14.41)	<0.01	31 (32.29)	52 (16.30)	<0.01
Giardia	17 (8.9)	11 (10.5)	37 (15.6)	00.01	6 (7.1)	30 (13.4)	0.13	34 (18.09)	13 (14.13)	98 (27.68)	<0.01	21 (21.88)	66 (20.69)	00.67

***Variable will be added in multiple models if p<0.25 in bi-variate model**
***All variables are adjusted for country**
¶ Mean ± standard deviation; Stunting: HAZ < −2, (%; for < 5 years of age), Underweight: WAZ < −2, (%; for < 5 years of age), Wasted: WHZ < −2, (%; for < 5 years of age), MUAC (mid upper arm circumference), median (mean; for < 5 years of age) Mid upper arm circumference, RBC = Red blood cell; Diarrhea: 3 or more stool/day, Fever: measured at least 38-degree c or parental perception; WASH; water, sanitation, and hygiene; OR, odds ratio; CI, confidence interval; SD., standard deviation; ND, not done; NTS, non-typhoidal *Salmonella;* MSD, Moderate to severe diarrhea

S2 Table. Overall demographics, housing, animal exposures, clinical presentation, stool examination, and co-pathogens of the NTS-positive under 5 children

Characteristics	NTS-positive n (%)	NTS- negative n (%)	p-value*
**Age group**			
0-11m	184 (48.6)	445 (39.2)	-
12-23m	119 (31.4)	395 (34.8)	0.02
24-59m	75 (19.8)	294 (25.9)	<0.001
**Gender (female)**	152 (40.2)	497 (43.8)	0.22
**Breastfed**	270 (71.4)	785 (69.2)	0.41
**Baseline Anthropometry**			
Wasted	65 (17.2)	150 (13.2)	0.05
Stunted	128 (34.0)	357 (31.5)	0.36
Underweight	126 (33.3)	303 (26.7)	0.01
MUAC (mean ± SD)	13.7 ± 1.5	14.0 ± 1.5	<0.001
**Clinical features**			
Visible blood in stool	55 (14.5)	134 (11.8)	0.16
Vomiting ≥3 times/ day	84 (22.2)	194 (17.1)	0.02
Fever	190 (50.2)	449 (39.6)	<0.001
Moderate to severe diarrhea	461 (40.6)	190 (50.2)	<0.001
**Socio-demographic characteristics**			
Primary caretaker (mother)	367 (97.0)	1107 (97.6)	0.57
Mother's education			
Illiterate	112 (29.6)	359 (31.7)	0.44
Household characteristics			
People regularly live in house (mean ± SD)	11.4 ± 9.0	9.4 ± 8.2	0.07
People regularly sleep in house (mean ± SD)	8.1 ± 9.1	9.2 ± 11.4	0.09
Presence of under 5 children at house (mean ± SD)	1.0 ± 1.4	0.9 ± 1.5	0.30
Natural floor (made of earth, sand, dung)	206 (54.5)	644 (56.7)	0.43
Wealth index			
Poorest	62 (16.4)	211 (18.6)	-
Lower middle	84 (22.2)	242 (21.3)	0.38
Middle	89 (23.5)	278 (24.5)	0.65
Upper middle	63 (16.6)	185 (16.3)	0.47
Richest	80 (21.1)	218 (19.2)	0.25
Animal at house			
Cow	167 (44.1)	443 (39.0)	0.07
Dog	197 (52.1)	557 (49.1)	0.31
Cat	196 (51.8)	554 (48.8)	0.31
Rodent/ Fowl	246 (65.0)	719 (63.4)	0.55
Goat	145 (38.3)	389 (34.3)	0.15
Sheep	80 (21.1)	232 (20.4)	0.76
**WASH**			
The main source of drinking water			
Use tube well water	81 (21.4)	263 (23.1)	0.47

Treat drinking water	151 (39.9)	426 (37.5)	0.40
Fecal disposal			
Toilet facility available	330 (87.3)	996 (87.83)	0.78
Hand washing practice			
Before eating	301 (79.6)	918 (80.9)	0.57
Before cooking	220 (58.2)	655 (57.7)	0.88
Before nurse/prepare baby food	128 (33.8)	378 (33.3)	0.85
After defecation	267 (70.6)	778 (68.6)	0.46
After handling animals	49 (12.9)	120 (10.5)	0.20
After cleaning a child	166 (43.9)	465 (41.0)	0.32
Hand wash material			
Routinely uses water and soap	298 (78.8)	933 (82.2)	0.13
**Stool examination**			
Loose watery stool	89 (23.5)	228 (20.1)	0.15
RBC present in stool	29 (7.6)	67 (5.91)	0.22
Pus present in stool	16 (4.2)	47 (4.1)	0.94
Mucus present in stool	147 (39.1)	350 (30.8)	<0.001
**Co pathogen isolated from stool**			
Campylobacter	65 (17.2)	175 (15.4)	0.41
Giardia	51 (13.4)	231 (20.3)	<0.001
Cryptosporidium	26 (6.8)	78 (6.8)	1.00
EAEC	78 (20.6)	227 (20.0)	0.79
ETEC	30 (7.9)	101 (8.9)	0.56

***All variables are adjusted for country**

Stunting: HAZ < −2, (%; for < 5 years of age), Underweight: WAZ < −2, (%; for < 5 years of age), Wasted: WHZ < −2, (%; for < 5 years of age), MUAC, median (mean; for < 5 years of age) Mid upper arm circumference, RBC = Red blood cell

Fever: measured at least 38-degree c or parental perception; WASH; water, sanitation, and hygiene

OR, odds ratio; CI, confidence interval; SD., standard deviation; NTS, non-typhoidal *Salmonella;* EAEC: Enteroaggregative *E. coli,* ETEC: Enterotoxigenic *E. coli.*
